# The Lipid Virulence Factors of *Mycobacterium tuberculosis* Exert Multilayered Control over Autophagy-Related Pathways in Infected Human Macrophages

**DOI:** 10.3390/cells9030666

**Published:** 2020-03-09

**Authors:** Aïcha Bah, Merlin Sanicas, Jérôme Nigou, Christophe Guilhot, Catherine Astarie-Dequeker, Isabelle Vergne

**Affiliations:** 1 Institut de Pharmacologie et de Biologie Structurale, Université de Toulouse, CNRS, Université Paul Sabatier, 31077 Toulouse, France; aicha.bah25@gmail.com (A.B.); merlinsanicas@gmail.com (M.S.); jerome.nigou@ipbs.fr (J.N.); christophe.guilhot@ipbs.fr (C.G.); 2 University of Lyon, Université Claude Bernard Lyon 1, 69100 Villeurbanne, France

**Keywords:** autophagy, mycobacterium, tuberculosis, lipids, macrophage, phagosome, lysosomes, innate immunity

## Abstract

Autophagy is an important innate immune defense mechanism that controls *Mycobacterium tuberculosis* (*Mtb*) growth inside macrophages. Autophagy machinery targets *Mtb*-containing phagosomes via xenophagy after damage to the phagosomal membrane due to the Type VII secretion system Esx-1 or via LC3-associated phagocytosis without phagosomal damage. Conversely, *Mtb* restricts autophagy-related pathways via the production of various bacterial protein factors. Although bacterial lipids are known to play strategic functions in *Mtb* pathogenesis, their role in autophagy manipulation remains largely unexplored. Here, we report that the lipid virulence factors sulfoglycolipids (SLs) and phthiocerol dimycocerosates (DIMs) control autophagy-related pathways through distinct mechanisms in human macrophages. Using knock-out and knock-in mutants of *Mtb* and *Mycobacterium bovis* BCG (Bacille Calmette Guerin) and purified lipids, we found that (i) *Mtb* mutants with DIM and SL deficiencies promoted functional autophagy via an MyD88-dependent and phagosomal damage-independent pathway in human macrophages; (ii) SLs limited this pathway by acting as TLR2 antagonists; (iii) DIMs prevented phagosomal damage-independent autophagy while promoting Esx-1-dependent xenophagy; (iv) and DIMs, but not SLs, limited the acidification of LC3-positive *Mtb* compartments. In total, our study reveals an unexpected and intricate role for *Mtb* lipid virulence factors in controlling autophagy-related pathways in human macrophages, thus providing further insight into the autophagy manipulation tactics deployed by intracellular bacterial pathogens.

## 1. Introduction

Each year, over 1.5 million people die from tuberculosis. This pulmonary infectious disease, which is caused by *Mycobacterium tuberculosis* (*Mtb*), remains the leading cause of death worldwide of bacterial origin. *Mtb* pathogenicity is closely related to its ability to survive in macrophages through the deployment of strategies to escape or neutralize host defenses [1]. One of these defenses is autophagy, a cellular process that allows for the capture of intracellular bacteria and their killing by lysosomes [2,3,4,5].

After phagocytosis by macrophages, *Mtb* resides in a single-membrane-bound compartment called the phagosome. The autophagy machinery targets *Mtb*-containing phagosomes via two main degradative pathways, xenophagy and LC3-associated phagocytosis (LAP) [6,7,8,9,10]. The LAP pathway is triggered upon bacterial phagocytosis (after the engagement of pattern recognition receptors (PRRs) on the cell surface) to promote LC3 conjugation to the phagosomal membrane: this phagosome is called a LAPosome [11]. Interestingly, *Mtb* RNA delivered by extracellular vesicles from infected macrophages in combination with IFN-β can also promote LAP via an RIG-I/MAVS-dependent pathway [12]. On the other hand, damage to the phagosomal membrane, which is triggered by the Type VII secretion system Esx-1, promotes xenophagy via the exposure of pathogen-associated molecular patterns (PAMPs) to the cytosolic sensor cGAS (cyclic GMP-AMP synthase), followed by STING signaling and the ubiquitination of damaged phagosome or via galectin recruitment onto the damaged phagosome [7,13,14,15]. Alternatively, the xenophagy of *Mtb*-containing phagosomes can be achieved exogenously using pharmaceutical drugs or immunomodulators, such as cytokines [5,16]. Xenophagy requires the formation of an LC3-decorated double-membrane-bound compartment, which is called an autophagosome and engulfs the bacteria. Importantly, both the LAP and xenophagy pathways result in acidification, fusion with lysosomes of the *Mtb* compartment, and eventually bacterial death [7,10].

*Mtb* evades various host defense mechanisms (autophagy is one of them) [1,5]. Over the past decade, several studies have identified the mycobacterial proteins implicated in autophagy inhibition. For instance, Eis, an N-acetyl transferase, and PE_PGRS47 prevent autophagy initiation, whereas Esx-1 limits autophagic flux, i.e., fusion with lysosomes [17,18,19]. Recently, CpsA has been shown to restrict the LAP pathway by impairing the assembly of the NADPH oxidase NOX2 [10]. While quite a few mycobacterial proteins have been investigated, very little is known about the role of *Mtb* lipids in macrophage autophagy. In particular, little information is available on the noncovalently associated lipids located in the outermost part of the cell envelope, despite the fact that several of them contribute efficiently to pathogenesis [20,21].

These complex lipids are present on the surface of the bacteria and can be trafficked inside infected cells, as well as extracellularly. Some of them are potent immunomodulators. They can act as ligands of toll-like receptor 2 (TLR2) and can trigger autophagy upon phagocytosis [22,23]. Others, such as mannose-capped lipoarabinomannan, limit LC3 recruitment onto latex beads containing phagosomes via an unknown mechanism [23,24]. A few envelope lipids also have a role in *Mtb* virulence, among which are major lipid virulence factors phthiocerol dimycocerosates (DIMs, also called PDIMs), and *Mtb*-specific lipids sulfoglycolipids (SLs, also called SGLs). Recently, DIMs have been proposed to induce autophagy. Indeed, THP-1 macrophages infected with an *Mtb* mutant with DIM transport deficiencies have lower levels of GFP-LC3 associated with their membranes than do those with wild-type (WT) *Mtb*, suggesting either a decrease in autophagy or an activation of LC3 degradation [25,26]. Whether DIMs promote the targeting of *Mtb* through autophagy machinery also remains to be investigated [26]. Another work has reported that purified *Mtb* SLs promote GFP-LC3 puncta accumulation in macrophages [27]. Again, it is unclear whether SLs activate autophagy or prevent LC3 turnover. In addition, the importance of SLs in the bacterial context has not been addressed. Collectively, these published data suggest that DIMs and SLs may modulate macrophage autophagy, but how these lipids precisely control autophagy-related pathways that target *Mtb*-containing phagosomes needs to be explored.

Here, we investigated the contributions of *Mtb* lipid virulence factors (DIMs and SLs) to the modulation of autophagy during the course of human macrophage infection. Our work unveiled a multifaceted role for *Mtb* lipid virulence factors in controlling autophagy-related pathways. Overall, this study deepens our understanding of autophagy manipulation by *Mtb* and the functions of lipid virulence factors in infection.

## 2. Materials and Methods

### 2.1. Antibodies and Reagents

The following rabbit antibodies were used: Atg16L1 (#PA1-18296 Thermo Scientific), beclin-1 (#sc-11427 Santa Cruz), and LC3 (#L7543 Sigma, #PM036 MBL). The following mouse antibodies were used: beta-actin (#sc-81178 Santa Cruz), galectin-3 (#556904 BD Pharmingen), and ubiquitin (FK2, #BML-PW8810 Enzo Life Sciences). Pam3CSK4 was purchased from Invivogen. The synthetic SL analog (2,3-dipalmitoyl-2′-sulfate-α-α′-d-trehalose) we used was a kind gift from Drs. Jacques Prandi and Martine Gilleron (Institute of Pharmacology and Structural Biology, Toulouse) [28,29]. The DIMs were prepared as previously described [30].

### 2.2. Bacterial Strains and Growth Conditions

The green fluorescent protein (GFP)-expressing strains used in this study included WT *Mtb* H37Rv Pasteur, *Mtb* H37RvΔ*ppsE*,Δ*pks2* (PMM127, deficient in DIMs and SLs), *Mtb* H37RvΔ*ppsE*,Δ*pks2::ppsE* (PMM127/DIM, complemented with DIM production), *Mtb* H37RvΔ*ppsE*,Δ*pks2::pks2* (PMM127/SL, complemented with SL production), WT *M. bovis* BCG Pasteur 1173P2, *M. bovis* BCG::*ESX-1*, BCGΔ*mas* (deficient in DIMs), and BCGΔ*mas*::*ESX-1* [31]. All strains were cultured at 37 °C in liquid Middlebrook 7H9 liquid medium (Difco) supplemented with 10% (*v*/*v*) ADC (albumin-dextrose-catalase, Difco) and 0.05% (*v*/*v*) Tween-80. When required, kanamycin and hygromycin were added to the medium at a final concentration of 40 μg/mL and 50 μg/mL, respectively.

### 2.3. Cell Culture and Infection

Human blood from fully anonymous nontuberculous donors was purchased from the Etablissement Français du Sang of Toulouse. Human macrophages derived from monocytes (hMDMs) were prepared as described in Reference [32]. In brief, monocytes plated into 24-well plates were isolated by adhesion from peripheral blood mononuclear cells (PBMCs) and allowed to differentiate for 7 days in RPMI (Roswell Park Memorial Institute) 1640 medium (Gibco, USA) supplemented with 2 mM glutamine (Gibco) and 7% (*v*/*v*) heat-inactivated human AB serum at 37 °C in 5% CO_2_. The culture medium was renewed on the third day. Before use, hMDMs were washed twice with fresh RPMI medium containing glutamine.

Human monocytic THP-1 cells (ATCC TIB-202T) and WT THP-1-Xblue^TM^ and THP-1-Xblue^TM^ MyD88 knockouts (Invivogen) were cultured in complete RPMI 1640 medium containing 10% (*v*/*v*) heat-inactivated fetal bovine serum, 2 mM L-Glutamine, 1 mM sodium pyruvate, and 1% (*v*/*v*) MEM (minimum essential media) nonessential amino acids. THP-1 monocytes were differentiated into macrophages with 20 ng/mL phorbol 12-myristate 13-acetate (PMA, Fisher bioreagents) for 24 h at 37 °C in 5% CO_2_. The PMA was washed away, and the cells were rested for at least 1 h in RPMI medium before infection.

A mycobacterial infection was performed as described in Reference [32]. Briefly, exponentially growing mycobacteria were pelleted by centrifugation and subsequently dispersed in serum-free RPMI 1640 medium using sterile glass beads. The number of bacteria per ml in the suspension was estimated through a measurement of the optical density at 600 nm. Human macrophages were infected for 1 h at an MOI (multiplicity of infection) between 5 and 10 in RPMI medium at 37 °C in 5% CO_2_. Extracellular bacteria were then removed by three successive washes with fresh medium, and infected cells were further incubated in RMPI medium supplemented with heat-inactivated serum at 37 °C in 5% CO_2_.

### 2.4. Western Immunoblotting and a Proteome Profiler Human Phospho-Kinase Array Kit

THP-1 cells were seeded and differentiated in 6-well plates (5 × 10^6^ cells). Cells were infected with *Mtb* strains at MOI = 10 for 1 h, and then extracellular bacteria were washed away. Infected cells were incubated for 48 h in the presence or absence of 100 nM Bafilomycin A1 (Santa Cruz, CA, USA) (for the last 2 h) (0.1% DMSO (dimethyl sulfoxide) was used as a negative control, Sigma, USA). For the Pam3CSK4 experiment, cells were treated with the indicated concentration for 2 h, and then 100 nM Bafilomycin A1 or the DMSO control was added and cells were incubated for 2 h to block autophagic flux and accumulate LC3-II. After incubation, the cells were lysed with a Laemmli buffer containing beta-mercaptoethanol and boiled for 10–15 min at 95 °C. Denaturated proteins were subjected to SDS (sodium dodecyl sulfate-polyacrylamide gel electrophoresis (4–16% gradient) using a Tris/glycine buffer system (BioRad, USA). After electrophoresis, proteins were transferred to a nitrocellulose transfer membrane. Blots were blocked with 5% dried milk or bovine serum albumin (BSA) in PBS (phosphate buffered saline) and then incubated with primary antibodies and the corresponding horseradish peroxidase-conjugated secondary antibody (Thermo Scientific, USA). Immunostaining was detected with SuperSignal West Pico Chemiluminescent Substrate (Thermo Scientific). A beta-actin band was used as a loading control. A proteome profiler array kit (ARY003B) was purchased from R&D Systems (USA). Each array contained two membranes spotted in duplicate with antibodies against 43 different phosphorylated proteins and 2 related total proteins. The assay was performed according to the manufacturer’s instructions. Membranes were visualized with a ChemiDoc^TM^ Touch Imaging System (BioRad), and integrated densities of Western blot bands and phospho-kinase array dots were measured using Image J software (National Institutes of Health).

### 2.5. Immunofluorescence, Lysotracker Staining, and Confocal Microscopy

Macrophages cultured on sterile glass coverslips were infected with GFP (green fluorescent protein)-expressing mycobacteria and fixed with 4% (*v*/*v*) paraformaldehyde (Electron Microscopy Sciences, USA) at different times after infection. Cells were then permeabilized for 5 min through a treatment in PBS containing 0.1% Triton X100. After blocking with 4% (*w*/*v*) BSA (bovine serum albumin) and 2% (*v*/*v*) goat serum in PBS, permeabilized cells were incubated with primary antibody followed by secondary Alexa 568-conjugated antibody or Alexa 647-conjugated antibody (Invitrogen, France). For the LysoTracker Red (LTR) experiment, cells were incubated during the last two 2 h of postinfection with LTR (Invitrogen) at 1 μM, followed by several washes and fixation. The coverslips were mounted onto glass slides with fluorescent mounting medium (Dako) and were analyzed on a Zeiss LSM510 or Olympus FV1000 confocal microscope. Around 15 to 30 random images were taken per condition per independent experiment. Images were analyzed using LSM510 or Image J software.

### 2.6. Statistical Analyses

The data were from at least three independent experiments, and they are shown as the mean ± SEM (standard error of the mean). Statistical analyses were performed with Graphpad Prism version 5 software using Student’s two-tailed *t*-test. Differences were considered significant when the *p*-value was inferior to 0.05.

## 3. Results

### 3.1. The Loss of SLs and DIMs Promoted LC3 Recruitment to M. tuberculosis Compartments

**Figure 1:**
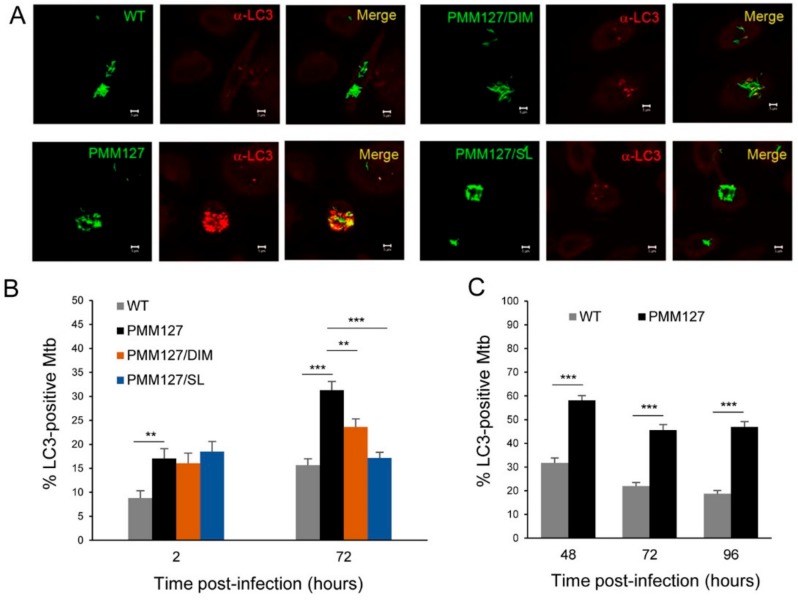
The loss of phthiocerol dimycocerosates (DIMs) and sulfoglycolipids (SLs) promoted LC3 recruitment to *Mycobacterium tuberculosis (Mtb)* compartments in human macrophages. Macrophages were infected for 1 h with the indicated GFP (green fluorescent protein)-expressing *Mtb* strains; fixed at 2 h, 48 h, 72 h, or 96 h postinfection (p.i.) (MOI (multiplicity of infection) between 5 to 10); permeabilized; incubated with antibody against endogenous LC3; and then stained with Alexa-568-labeled secondary antibody. Specimens were analyzed using confocal fluorescence microscopy. (**A**) Representative confocal images of hMDMs (human monocyte derived macrophages) infected with GFP-expressing *Mtb* (green channel) at 72 h postinfection, stained for endogenous LC3 (red channel). Scale bar, 5 μm. (**B**) A quantification of the percentage of *Mtb* compartments colocalized with LC3 at 2 h and 72 h postinfection in hMDMs. Data are the mean ± s.e.m (standard error of mean); 160–200 fields from four independent experiments; ** *p* < 0.01; *** *p* < 0.001 (unpaired *t*-test). (**C**) A quantification of the percentage of *Mtb* compartments colocalized with LC3 at 48 h, 72 h, and 96 h postinfection in PMA (phorbol 12-myristate 13-acetate)-differentiated THP-1 macrophages. Data are the mean ± s.e.m; 90 fields from three independent experiments; *** *p* < 0.001 (unpaired *t*-test). DIMs: phthiocerol dimycocerosates; SLs: sulfoglycolipids; WT: H37Rv wild-type; GFP: green fluorescent protein; hMDMs: human monocyte-derived macrophages; PMM127: H37RvΔ*ppsE*,Δ*pks2*; PMM127/DIMs: H37RvΔ*ppsE*,Δ*pks2*::*ppsE*; PMM127/SLs: H37Rv Δ*ppsE*,Δ*pks2*::*pks2*.

In our previously published work, we reported that the *Mtb* mutant PMM127, which is lacking in both DIMs and SLs, is trafficked more efficiently to lysosomes than is WT *Mtb* (in human macrophages) [33]. Importantly, the *Mtb* mutant PMM127 does not represent a significant change to the bacterial cell envelope structure [33]. However, whether this *Mtb* mutant interacts differently with autophagy machinery than does WT *Mtb* was not investigated. First, we examined endogenous LC3’s association with the *Mtb* intracellular compartment using immunofluorescence and confocal microscopy. Human monocyte-derived macrophages (hMDMs) were pulsed with GFP-expressing *Mtb* strains for 1 h, chased for different time points, and stained with a specific antibody against LC3. At both early (2 h) and late time points (72 h) postinfection (p.i.), the percentage of *Mtb* positive for LC3 was two times higher with PMM127 than with WT (Figure 1A,B). These results were also observed in PMA-differentiated THP-1 macrophages up to 96 h p.i (Figure 1C). To determine the contribution of each type of lipid to the inhibition of LC3 recruitment, macrophages were infected with PMM127 that had been complemented genetically for either DIM or SL production [31]. At 2 h p.i., the complemented strains behaved like PMM127. However, at a late time point p.i. (72 h), DIM and SL production significantly reduced LC3 colocalization with PMM127-containing compartments by 50% and 90%, respectively (Figure 1A,B). These results reveal that *Mtb* producing SLs, and to a lesser extent DIMs, are able to prevent LC3 recruitment to *Mtb* compartments in human macrophages.

### 3.2. The Mtb Mutant Deficient in DIMs and SLs Promoted Autophagy Activation

**Figure 2:**
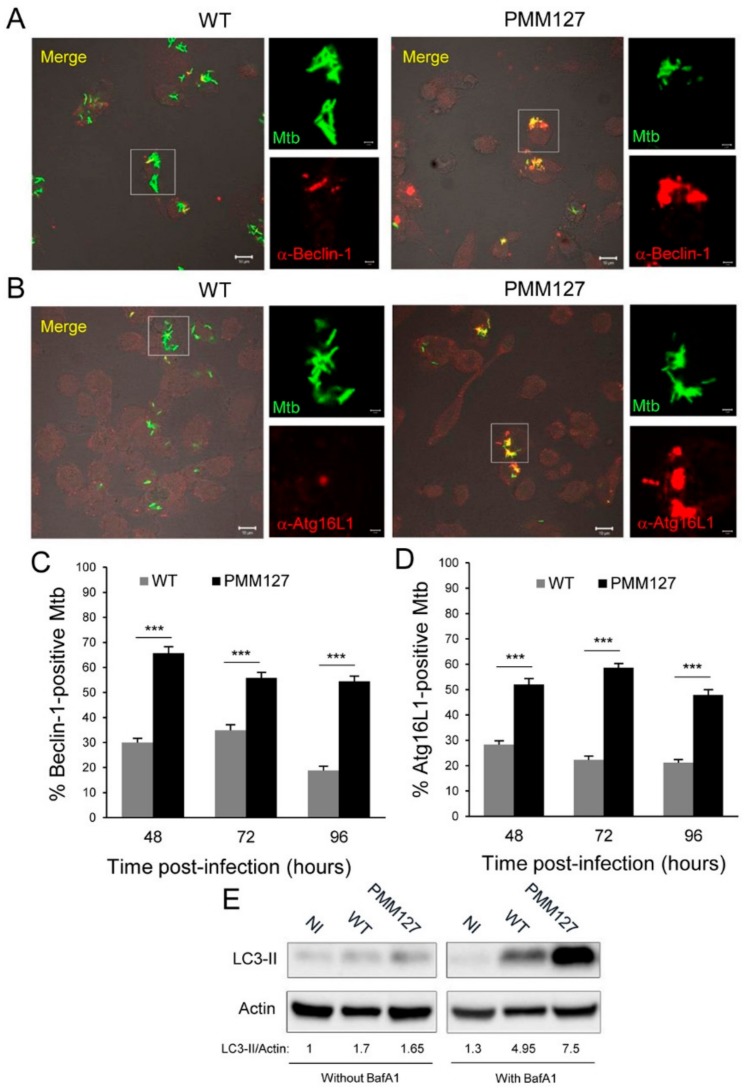
The *Mtb* mutant deficient in DIMs and SLs promoted autophagy activation. (**A**–**D**) Differentiated THP-1 cells were infected for 1 h with the indicated GFP-expressing *Mtb* strains; fixed at 48 h, 72 h, or 96 h postinfection (MOI between 5 and 10); permeabilized; incubated with antibodies against endogenous Beclin-1 (**A**,**C**) or Atg16L1 (**B**,**D**); and then stained with Alexa-568-labeled secondary antibody. Specimens were analyzed using confocal fluorescence microscopy. (**A**,**B**) Representative confocal images of macrophages infected with GFP-expressing *Mtb* (green channel) at 48 h postinfection, stained for Beclin-1 (**A**) or Atg16L1 (**B**) (red channel). The boxed areas in the left-hand panels are magnifications of the right-hand panels. Scale bars are 10 μm (left panels) or 2 μm (right panels). (**C**,**D**) A quantification of the percentage of *Mtb* compartments colocalized with Beclin-1 (**C**) or Atg16L1 (**D**) at 48 h, 72 h, and 96 h postinfection. Data are the mean ± s.e.m; 90 fields from three independent experiments; *** *p* < 0.0001 (unpaired *t*-test). (**E**) Differentiated THP-1 cells were infected for 1 h with the indicated *Mtb* strains and lysed 48 h postinfection. Cells were treated with 100 nM Bafilomycin A1 (with BafA1) or a DMSO (dimethylsulfoxide) (control (without BafA1) during the last 2 h postinfection. Lysates were analyzed using immunoblotting with anti-LC3 or anti-actin antibodies. The densitometric LC3-II/actin ratios are shown underneath the blot. The ratios were normalized to the ratio of noninfected (NI) cells in the absence of BafA1. Data are representative of two independent experiments.

Several studies have revealed that LC3 can be involved in autophagy-independent processes [34]. To confirm that PMM127 is more targeted by autophagy machinery than is WT *Mtb*, we examined the intracellular localization of endogenous Beclin-1 and Atg16L1, two autophagy-related gene (Atg) products acting upstream of LC3. An immunofluorescence confocal microscopy analysis and quantification showed that around 65% to 50% of PMM127 was colocalized with Beclin-1 and Atg16L1 independently of the time postinfection, whereas only 30% to 20% of WT *Mtb* was associated with these proteins (Figure 2A–D). To determine whether the increase in Atg recruitment was associated with an upregulation of these proteins, we evaluated the protein levels of Beclin-1 and Atg16L1 through immunoblotting. Infection with PMM127 induced an approximately two-fold increase in the Beclin-1 protein amount compared to WT *Mtb* and noninfected macrophages (Figure S1A,B). Although not significant, a similar trend was observed for Atg16L1 (Figure S1C,D). Using quantitative RT-PCR, we found that both WT *Mtb* and PMM127 induced an increase in *BECN1* gene expression, but not in *ATG16L1* expression (Figure S1E,F). However, no difference was detected between the two strains for either gene, indicating that the difference observed upon infection with WT or PMM127 was not due to differential gene expression. Collectively, these data indicate that the lipid-deficient *Mtb* mutant promoted autophagy machinery recruitment to bacterial compartments.

To further explore autophagy activation, LC3-II lysosomal turnover was determined using Bafilomycin A1 (BafA1), an inhibitor of lysosomal degradative activities, and immunoblotting. WT *Mtb* and PMM127 infections resulted in an increase in LC3-II turnover compared to noninfected macrophages (Figure 2E). However, the turnover was almost two-fold higher with PMM127 (LC3-II/actin with BafA1 minus LC3-II/actin without BafA1 = 5.85) than with WT (LC3-II/actin with BafA1 minus LC3-II/actin without BafA1 = 3.25) (Figure 2E). Furthermore, in the presence of BafA1, the total level of LC3-II was much greater with PMM127 than with the WT, which supported the idea of the activation of autophagy initiation. Altogether, these results indicate that the lipid-deficient *Mtb* mutant unleashed functional autophagy in macrophages.

### 3.3. The Mtb Mutant Deficient in DIMs and SLs Promoted Autophagy via MyD88

In order to get some insight into the signaling pathway involved in autophagy activation induced by PMM127, we used an unbiased approach based on a phosphoprotein-specific antibody array [35]. We found that the two major signaling pathways implicated in autophagy regulation, i.e., mTOR (mammalian target of rapamycin) (p70S6K phosphorylation) and AMPK (5′ AMP-activated protein kinase), were activated in the WT *Mtb*- and PMM127-infected macrophages (Figure S2A,B) [36]. However, the levels of activation were similar between WT and PMM127, indicating that these two pathways were not responsible for the autophagy upregulation observed with *Mtb* mutants deficient in DIMs and SLs.

**Figure 3:**
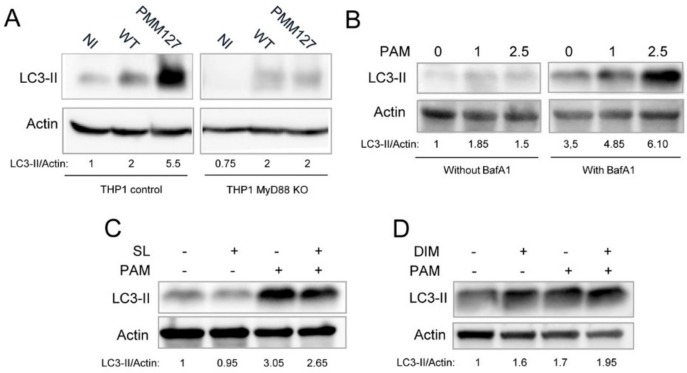
Synthetic SL, but not purified DIM, molecules limited TLR2-dependent autophagy. (**A**) Differentiated THP-1 cells, either wild-type (control) or MyD88 knockouts (KOs), were infected for 1 h with the indicated *Mtb* strains (WT and PMM127) and lysed 48 h postinfection. Cells were treated with 100 nM Bafilomycin A1 (BafA1) during the last 2 h post-infection. Lysates were analyzed using immunoblotting with anti-LC3 or anti-actin antibodies. The densitometric LC3-II/actin ratios are shown underneath the blot. The ratios were normalized to the ratio of noninfected (NI) cells. Data are representative of two independent experiments. (**B**) Differentiated THP-1 cells were treated with different concentrations (μg/mL) of Pam3CSK4 (PAM) for 2 h, and then 100 nM bafilomycin A1 (BafA1) or a DMSO control was added for 2 h more. Lysates were analyzed using immunoblotting with anti-LC3 or anti-Actin antibodies. LC3-II/actin ratios were normalized to the ratio of cells without Pam3CSK4 and BafA1. (**C**) Differentiated THP-1 cells were pretreated for 30 min with 10 μg/mL of SL synthetic analog and then incubated with 2.5 μg/mL Pam3CSK4 for 2 h. Then, 100 nM bafilomycin A1 (BafA1) was added for 2 h more. LC3-II/actin ratios were normalized to the ratio of cells without Pam3CSK4. Data are representative of two independent experiments. (**D**) Differentiated THP-1 cells were pretreated for 60 min with 70 μM (100 μg/mL) of purified DIMs and then incubated with 2.5 μg/mL Pam3CSK4 for 2 h. Then, 100 nM bafilomycin A1 (BafA1) was added for 2 h more. LC3-II/actin ratios were normalized to the ratio of cells without Pam3CSK4. Data are representative of two independent experiments.

The TLR/MyD88 signaling pathway is known to promote autophagy [3], in particular in mycobacteria-infected macrophages [22,37]. Therefore, we investigated its role in PMM127-induced autophagy. Differentiated THP-1 macrophages (WT or MyD88 knockouts (KOs)) were infected with WT *Mtb* or PMM127 and treated with BafA1 to determine the total amount of LC3-II formed upon infection. Immunoblot analyses showed that the LC3-II level in infected cells was reduced in MyD88-deficient macrophages compared to WT macrophages (Figure 3A). Importantly, while in WT macrophages PMM127 induced more LC3-II than did WT *Mtb*, this difference was drastically reduced in MyD88-deficient macrophages. These results indicated that *Mtb* mutants deficient in DIMs and SLs promoted autophagy via TLR/MyD88. This is consistent with published data showing that SLs limit the TLR/MyD88 pathway in macrophages by acting as a competitive TLR2 antagonist [28]: it is also consistent with a recent report indicating that DIMs evade TLR signaling in macrophages [38], although the mechanism of action remains unclear [39].

### 3.4. Synthetic SL, but not Purified DIM, Molecules Limited TLR2-Dependent Autophagy

TLR2, one of the main PRRs (pattern recognition receptors) that recognize *Mtb* [40], has been implicated in autophagy activation in the context of mycobacterial infection [22,41,42]. Next, we investigated whether SLs and DIMs could per se limit TLR2-dependent autophagy by testing purified SL and DIM molecules. TLR2-dependent autophagy was induced by incubating macrophages with a synthetic lipopeptide TLR2 ligand, Pam3CSK4. First, we determined the concentration of Pam3CSK4 required for autophagy activation in THP-1 macrophages and found that 2.5 μg/mL was necessary to obtain about a two-fold increase in the LC3-II total in the presence of BafA1 (Figure 3B). Cells were then pretreated with a synthetic analog of SLs [28] or with purified DIMs, followed by Pam3CSK4 treatment in the presence of BafA1. We found that synthetic SLs at 10 μg/mL inhibited TLR2-mediated autophagy by around 20% but had a negligible effect on basal autophagy (Figure 3C). Similar results were obtained with SLs at 20 μg/mL (Figure S3A,C) and another TLR2 ligand, zymosan (Figure S3B). In contrast, purified DIMs at 100 μg/mL did not inhibit Pam3CSK4-induced autophagy, but rather seemed to activate basal autophagy (Figure 3D, Figure S3D). These results show that SL, but not DIM, molecules could directly prevent TLR2-mediated autophagy.

### 3.5. Autophagy Mediated by a Lipid-Deficient Mtb Mutant Was Not Associated with Phagosomal Damage

**Figure 4:**
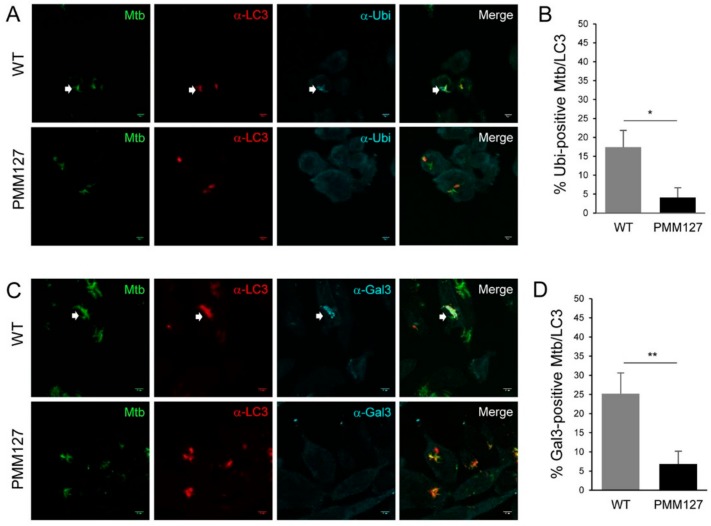
Autophagy mediated by a lipid-deficient *Mtb* mutant was not associated with ubiquitin coating or galectin-3 recruitment. Differentiated THP-1 cells were infected for 1 h with the indicated GFP-expressing *Mtb* strains (MOI = 5), fixed 72 h postinfection, permeabilized, incubated with antibodies against endogenous LC3 and ubiquitin (Ubi) or galectin-3 (Gal3), and then stained with Alexa-568- and Alexa-647-labeled secondary antibodies. Specimens were analyzed using confocal fluorescence microscopy. (**A**,**C**) Representative confocal images of macrophages infected with GFP-expressing *Mtb* (green channel) and stained for LC3 (red channel) and ubiquitin or galectin-3 (cyan channel). White arrows indicate *Mtb* compartments positive for LC3 and ubiquitin (**A**) or galectin-3 (**C**). Scale bar, 5 μm. (**B**,**D**) Quantification of the percentage of *Mtb* compartments colocalized with LC3 that were positive for ubiquitin (**B**) or galectin-3 (**D**). Data are the mean ± s.e.m; 45–55 fields from three independent experiments; * *p* < 0.05; ** *p* < 0.01 (unpaired *t*-test).

TLR/MyD88 is involved in autophagy pathways that target either damaged or intact phagosomes [37,43,44]. Since *Mtb* can reside in both types of phagosome, we examined whether enhanced LC3 recruitment was associated with enhanced phagosomal membrane damage using ubiquitin and galectin-3 as markers [45]. Immunofluorescence and confocal microscopy analyses showed that, at 3 days p.i., a small percentage of LC3-positive compartments containing WT *Mtb*, around 20%, were positive for ubiquitin and galectin-3 (Figure 4A–D). This result highlights the coexistence of two autophagy-related pathways targeting *Mtb*, one linked to phagosomal damage and a second that targets intact phagosomes. Importantly, the percentage of LC3 compartments containing *Mtb* that were positive for ubiquitin and galectin-3 dropped significantly to approximately 5% with PMM127. These findings indicate that the loss of DIMs and SLs did not enhance LC3 recruitment to the damaged phagosome, but rather promoted LC3 recruitment to the damage-free phagosome.

### 3.6. DIMs Limited Phagosomal Damage-Independent Autophagy while Promoting Esx-1-Dependent Autophagy

**Figure 5:**
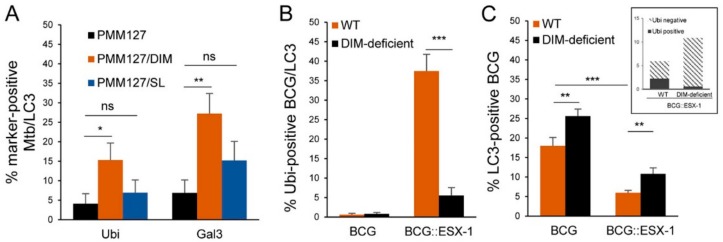
DIMs limited phagosomal damage-independent autophagy while promoting Esx-1-dependent autophagy. (**A**) Quantification of the percentage of *Mtb* compartments colocalized with LC3 that were positive for ubiquitin or galectin-3. Differentiated THP-1 cells were infected for 1 h with the indicated GFP-expressing *Mtb* strains (MOI = 5), fixed 72 h postinfection, permeabilized, incubated with antibodies against endogenous LC3 and ubiquitin (Ubi) or galectin-3 (Gal3), and then stained with Alexa-568- and Alexa-647-labeled secondary antibodies. Specimens were analyzed using confocal fluorescence microscopy. Data are the mean ± s.e.m; 45–55 fields from three independent experiments; * *p* < 0.05; ** *p* < 0.01 (unpaired *t*-test). (**B**,**C**) Here, hMDMs were infected for 1 h with the indicated GFP-expressing BCG (Bacille Calmette Guerin) strains (MOI 5), fixed 72 h postinfection, permeabilized, incubated with antibodies against endogenous LC3 and ubiquitin (Ubi), and then stained with Alexa-568- and Alexa-647-labeled secondary antibodies. Specimens were analyzed using confocal fluorescence microscopy. (**B**) Quantification of the percentage of BCG compartments colocalized with LC3 that were positive for ubiquitin. Data are the mean ± s.e.m; 45 fields from three independent experiments. (**C**) Quantification of the percentage of BCG compartments colocalized with LC3. Data are the mean ± s.e.m; 90 fields from three independent experiments. The inset is a repartition of LC3-positive/ubiquitin-positive and LC3-positive/ubiquitin-negative for BCG::Esx-1 wild-type (WT) or a Δmas mutant (DIM-deficient). * *p* < 0.05; ** *p* < 0.01; *** *p* < 0.001; ns: not significant (unpaired *t*-test).

As shown in Figure 4, we noted a higher proportion of damage-associated autophagy with WT *Mtb* than with a lipid-deficient mutant. To determine which lipids, DIMs or SLs, were involved in this process, macrophages were infected with PMM127 complemented genetically for DIM or SL production, and the percentage of LC3 compartments containing *Mtb* that were positive for ubiquitin or galectin-3 was counted. *Mtb* complementation for DIM production (but not SL) significantly increased the percentage of LC3-positive compartments containing *Mtb* that were positive for ubiquitin and for galectin-3 (Figure 5A). These results were consistent with our published data, which showed an increase in phagosomal damage when *Mtb* was complemented for DIM (but not for SL) production [31]. Thus, DIMs seemed to favor LC3 associated with phagosomal damage (Figure 5A). However, overall, DIMs inhibited LC3 recruitment to *Mtb* compartments (Figure 1A,B), suggesting that these lipids may inhibit LC3 recruitment to damage-free phagosomes. To validate this hypothesis, we investigated the respective contribution of these two pathways using *Mycobacterium bovis* Bacillus Calmette-Guérin (BCG), which is naturally deficient in SLs; Esx-1, a secretion system required for phagosomal damage; and recombinant BCG strains expressing different combinations of DIMs and Esx-1 [31]. Macrophages were infected with different GFP-expressing BCG strains and stained for endogenous LC3 and ubiquitin. As expected from a previous work [7], damage-dependent autophagy was induced by Esx-1, as seen in the occurrence of ubiquitin in LC3-positive compartments containing BCG::Esx-1 but not BCG (Figure 5B). Furthermore, we observed that DIM-deficient BCG::Esx-1 was less able to promote ubiquitin-associated autophagy than was DIM-proficient BCG::Esx-1. This confirmed that DIMs promoted damage-dependent autophagy in the presence of functional Esx-1. Nevertheless, the percentage of mycobacteria positive for LC3 was significantly higher in the absence of DIM, even in the absence of Esx-1, indicating that DIMs inhibited damage-independent autophagy (Figure 5C) while promoting Esx-1-dependent autophagy (Figure 5B).

### 3.7. DIMs, but not SLs, Limited the Acidification of LC3-Positive Compartments Containing Mtb

**Figure 6:**
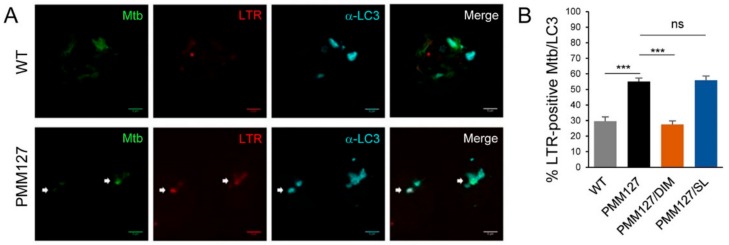
DIMs, but not SLs, limited the acidification of LC3^+^-compartment-containing *Mtb*. Differentiated THP-1 cells were infected for 1 h with the indicated GFP-expressing *Mtb* strains (MOI = 5) and fixed 72 h postinfection. Cells were incubated with LysoTracker Red (LTR) during the last 2 h postinfection. Fixed cells were then permeabilized, incubated with antibodies against endogenous LC3, and stained with Alexa-647-labeled secondary antibody. Specimens were analyzed using confocal fluorescence microscopy. (**A**) Representative confocal images of macrophages infected with GFP-expressing *Mtb* (green channel), labeled with LysoTracker Red (LTR, red channel) and stained with LC3 (cyan channel). White arrows indicate acidified LC3-positive *Mtb* compartments. Scale bar, 5 μm. (**B**) Quantification of the percentage of *Mtb* compartments colocalized with LC3 that were positive for LTR. Data are the mean ± s.e.m; 60 fields from three independent experiments; *** *p* < 0.001; ns: not significant (unpaired *t*-test).

The autophagy machinery of Beclin-1, Atg16L1, and LC3 targeted *Mtb* mutants deficient in DIMs and SLs (Figure 1 and Figure 2). To determine whether the *Mtb*-containing compartments positive for LC3 could be acidified, infected macrophages were stained with LysoTracker Red (LTR) and analyzed using immunofluorescence confocal microscopy [22]. At 72 h p.i., while only 30% of WT *Mtb* within the LC3-positive compartments was colocalized with LTR, we observed that more than 50% of the LC3-positive compartments containing PMM127 were acidified (Figure 6A,B), indicating that PMM127 not only enhanced LC3 recruitment compared to the WT (Figure 1) but also increased the acidification of LC3-positive bacterial compartments. To determine the contribution of each type of lipid to preventing the acidification of LC3-positive compartments containing *Mtb*, macrophages were infected with PMM127 complemented genetically for either DIM or SL production. Only 30% of PMM127/DIMs within the LC3-positive compartment were acidified compared to 50% with PMM127/SLs, indicating that DIM, but not SL, synthesis limited the acidification of mycobacterial compartments and restored the *Mtb* WT phenotype (Figure 6B).

## 4. Discussion

**Figure 7:**
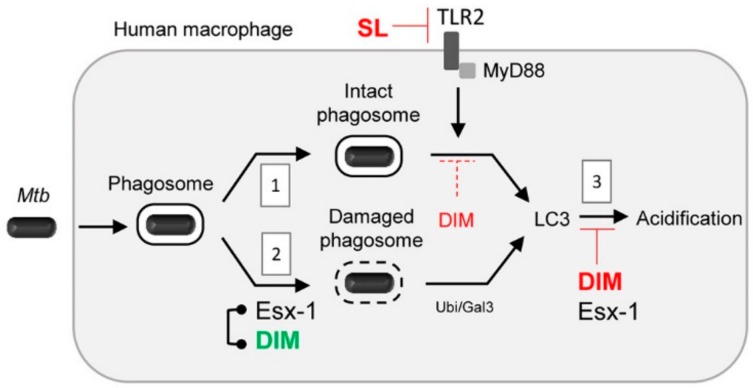
Schematic representation of SL and DIM action in autophagy-related pathways in *Mtb*-infected human macrophages. *Mtb* resides inside either a damaged or an intact phagosome [1]. The Type VII secretion system Esx-1 promotes phagosomal damage, which leads to xenophagy [7]. However, Esx-1 can also inhibit autophagic flux via an unknown mechanism [19,51,67]. Here, we found that (i) SLs limited TLR/MyD88-dependent and phagosomal damage-independent autophagy by acting as a TLR2 antagonist (**1**). (ii) DIMs prevented, to some extent, phagosomal damage-independent autophagy (dotted line) (**1**) while triggering xenophagy by favoring Esx-1-dependent phagosomal damage (**2**). (iii) Finally, DIMs, but not SLs, limited the acidification of LC3-positive compartments containing *Mtb* (**3**). Ubi: ubiquitin; Gal3: galectin-3. In terms of colors, autophagy modulation by SLs and DIMs is shown in red (inhibition) and green (activation).

In recent years, an increasing number of studies have reported that *Mtb* can circumvent autophagy pathways in macrophages by producing different bacterial proteins [10,17,18]. The present study reports, for the first time, that *Mtb* lipid virulence factors, namely DIMs and SLs, control autophagy at multiple levels in infected human macrophages. First, we found that *Mtb* mutants deficient in DIMs and SLs upregulated autophagy pathways that rely on MyD88 but not on phagosomal damage. SLs prevented this pathway by acting as an TLR2 antagonist. DIMs also limited autophagy independently of phagosomal damage, but in addition promoted an autophagy pathway that relied on Esx-1-mediated phagosomal damage. Finally, the presence of DIMs, unlike SLs, prevented acidification of the LC3-positive compartments containing *Mtb* (Figure 7).

Our previous work indicated that *Mtb* deficient in DIMs and SLs was attenuated in macrophages and in a mice model of infection (compared to WT *Mtb*) [33]. Furthermore, using an autophagy inhibitor, Bafilomycin A1, we were able to restore the WT *Mtb* growth phenotype [31], indicating that autophagy could contribute to growth attenuation. Here, we show that *Mtb* mutants deficient in DIMs and SLs promoted an autophagy-related pathway that required MyD88 but did not involve phagosomal damage. The LAP pathway is triggered upon phagocytosis after the engagement of PRRs on the cell surface (which includes TLR2) to promote LC3 conjugation in undamaged phagosomal membranes [44]. MyD88 is an important TLR adaptor protein that is involved in autophagy activation during the course of mycobacterial infection [37]. Recently, the LAP has been shown to target *Mtb* phagosomes [11], suggesting that *Mtb* mutants deficient in DIMs and SLs may stimulate a LAP process that involves TLR/MyD88 signaling. However, we cannot rule out the possibility that intact *Mtb* phagosomes might be captured in autophagosomes by a yet undefined molecular mechanism [46].

A growing amount of literature has demonstrated the key role of TLR/MyD88 signaling in bacterial infections [2,3,37,47]. *Mtb* produces numerous TLR ligands involved in triggering immune responses, but it has also developed several strategies to avoid recognition or to alter downstream signaling cascades [40]. Here, we found that SLs were able to restrict LC3 recruitment to an intact phagosome containing *Mtb*. As anticipated from our previous published data, which showed that SLs act as an antagonist of TLR2 [28], synthetic SLs prevented TLR2-induced autophagy. In contrast, purified DIMs did not prevent TLR2-mediated autophagy. This could be explained by either a requirement for the entire bacteria or the inhibition of MyD88-induced autophagy (which is independent of TLR2). In human macrophages, DIMs are transferred to host cell membranes and disorganize the lipid bilayer in a way that modulates phagocytic receptor functions [30,32]. We thus suspect that DIMs’ effects on membranes can affect membrane receptors such as TLR. Recently, the *Mtb* lipoprotein LprE has been shown to suppress TLR2-dependent autophagy [42]. Since *Mtb* produces several factors that antagonize TLR2, such as Esat-6 and phenolic glycolipids [48,49], it is tempting to speculate that these factors might also participate in limiting TLR2-induced autophagy during the course of *Mtb* infection.

Recently, DIMs, but not SLs, have been reported to be required for phagosomal damage and rupture in human macrophages [26,31]. Furthermore, a flow cytometry analysis has shown that *Mtb* mutants deficient in DIMs accumulated less membrane-bound LC3-II than did WT *Mtb*, which suggested reduced autophagy initiation, although this could also be interpreted as an increase in autophagic flux [26]. Along the same lines, in lymphatic endothelial cells, DIMs promote phagosomal rupture and recruitment of the xenophagy receptors NDP52 and p62 to *Mtb,* hinting toward a pro-xenophagy role for DIMs [50]. As anticipated, our findings demonstrate that DIMs, but not SLs, triggered the xenophagy of *Mtb* in human macrophages. Importantly, we found that DIMs induced xenophagy only when the mycobacteria expressed functional Esx-1, which underscores the significance of the interplay between DIM and Esx-1 in modulating host defense mechanisms, including autophagy.

Another interesting finding is the role of DIMs in preventing the acidification of LC3-positive *Mtb* compartments (Figure 7). *Mtb* seems to limit autophagic flux by preventing Rab7 recruitment in autophagosomal membranes [51]. One major *Mtb* factor known to block the last stage of autophagy is Esx-1 and its effector Esat-6, albeit their molecular mechanisms of action remain to be elucidated [19,51]. We have previously shown that DIMs prevent the acidification of the *Mtb* phagosome inside human macrophages [32]. Here, we found that DIMs also interfered with the acidification of LC3-positive compartments containing *Mtb*. How DIMs block the acidification of phagosomes is under intensive investigation. Since DIMs and Esx-1 synergize to promote phagosomal damage and xenophagy, it will be interesting to determine whether they also act together in preventing the acidification of autophagic compartments. Importantly, DIM action seems to be specific, as we did not observe such inhibition with SLs, even though purified SLs can modulate phagosome acidification [52,53]. Other mycobacterial lipids are known to prevent phagosome acidification [23,54,55,56,57]. Whether such lipids impair the acidification of autophagosomes or LAPosomes needs to be explored.

Compelling evidence has shown that DIMs and SLs exert multiple activities during the infection of macrophages, including the modulation of immune responses [20,26,28,31,38]. Here, we established a new role for DIMs and SLs in the control of autophagy. To survive in the adverse environment of the host, *Mtb* alters its gene expression. It should be noted that the genes involved in the biosynthesis of SLs and DIMs are upregulated upon infection [58,59]. In addition, the mass of these lipids increases as a result of their synthesis being coupled to cholesterol metabolism [60,61,62]. Given the multifaceted functions of SLs and DIMs, it is therefore reasonable to propose that the expansion of lipid pools in *M. tuberculosis* has important consequences in pathogenesis.

To conclude, our work uncovered novel *Mtb* virulence factors involved in the evasion of macrophage autophagy. To our knowledge, DIMs and SLs are the first bacterial lipids reported to modulate autophagy in an infection context. In addition to being attractive potential therapeutic targets in curtailing *Mtb* infection, DIMs and SLs, or synthetic functional analogs, might be useful tools for further deciphering the mechanisms of autophagy inhibition. Exploring these molecular mechanisms should help in the development of new compounds to either stimulate autophagy in the context of host-directed therapy against tuberculosis [5] or to limit detrimental autophagy in some other pathological contexts [63,64]. A deeper understanding of the multiple roles of autophagy in *Mtb* infection is warranted to discover important clues for the development of autophagy-based vaccines against and therapies for tuberculosis [65,66].

## Supplementary Materials

The following are available online: https://www.mdpi.com/2073-4409/9/3/666/s1, Figure S1: Levels of Beclin-1 and Atg16L1 in Mtb-infected macrophages, Figure S2: Status of mTOR and AMPK pathways in Mtb-infected macrophages, Figure S3: Synthetic SL but not purified DIM molecules limit TLR2-dependent autophagy.
